# Long-term changes in the small-world organization of brain networks after concussion

**DOI:** 10.1038/s41598-021-85811-4

**Published:** 2021-03-25

**Authors:** N. W. Churchill, M. G. Hutchison, S. J. Graham, T. A. Schweizer

**Affiliations:** 1 Keenan Research Centre for Biomedical Science, St. Michael’s Hospital, Toronto, ON Canada; 2 Neuroscience Research Program, St. Michael’s Hospital, Toronto, ON Canada; 3 Faculty of Kinesiology and Physical Education, University of Toronto, Toronto, ON Canada; 4 Physical Sciences Platform, Sunnybrook Research Institute, Sunnybrook Health Science Center, Toronto, ON Canada; 5 Department of Medical Biophysics, University of Toronto, Toronto, ON Canada; 6 Faculty of Medicine (Neurosurgery), University of Toronto, Toronto, ON Canada; 7 The Institute of Biomaterials and Biomedical Engineering (IBBME), University of Toronto, Toronto, ON Canada

**Keywords:** Neurological disorders, Network models

## Abstract

There is a growing body of literature using functional MRI to study the acute and long-term effects of concussion on functional brain networks. To date, studies have largely focused on changes in pairwise connectivity strength between brain regions. Less is known about how concussion affects whole-brain network topology, particularly the “small-world” organization which facilitates efficient communication at both local and global scales. The present study addressed this knowledge gap by measuring local and global efficiency of 26 concussed athletes at acute injury, return to play (RTP) and one year post-RTP, along with a cohort of 167 athletic controls. On average, concussed athletes showed no alterations in local efficiency but had elevated global efficiency at acute injury, which had resolved by RTP. Athletes with atypically long recovery, however, had reduced global efficiency at 1 year post-RTP, suggesting long-term functional abnormalities for this subgroup. Analyses of nodal efficiency further indicated that global network changes were driven by high-efficiency visual and sensorimotor regions and low-efficiency frontal and subcortical regions. This study provides evidence that concussion causes subtle acute and long-term changes in the small-world organization of the brain, with effects that are related to the clinical profile of recovery.

## Introduction

Concussion is a form of mild traumatic brain injury (mTBI) that is highly prevalent, with over 4 million cases occurring annually in North America^1^. Despite an absence of overt structural brain damage, it is associated with acute cognitive, somatic and affective disturbances, and approximately 10–25% of cases have effects lasting months to years after injury^2,3^. In the sport context, concussion diagnosis is based on brief assessments of symptom status, cognition and balance. Medical clearance to return to play (RTP) is also determined by symptom resolution, following the completion of a graded exercise protocol^4^. However, standard clinical assessments only indirectly reflect the underlying injury, and there is concern that biological recovery lasts beyond symptom resolution, with long-term physical and mental health consequences that may be further exacerbated by multiple concussions^5,6^. It is therefore an ongoing challenge to characterize concussion pathophysiology and to determine whether recovery processes continue beyond medical clearance to RTP.

Functional neuroimaging studies have established that disrupted communication between brain regions plays a key role in post-concussion impairments^7–10^. Blood-oxygenation-level dependent functional MRI (BOLD fMRI) has been widely used to evaluate post-concussion changes in brain function, based on fluctuations in blood oxygen levels. These studies have identified post-concussion alterations in functional connectivity, by measuring the temporal synchronization of BOLD signals between brain regions. To date, most studies have focused on changes in the pairwise connectivity strength of pre-defined brain regions, with the default mode network (DMN) being a popular target^8,11–13^. But concussive injuries are known to be highly variable between individuals in terms of injury biomechanics^14^, with correspondingly heterogeneous and spatially diffuse patterns of microstructural brain injury^15,16^. This suggests a need for functional connectivity analyses that account for the complex and spatially distributed changes in brain function that are associated with injury and recovery^17^.

An elegant method of addressing this need is through graph theory, which treats brain networks as graph structures. Powerful concepts have been developed in graph theory to describe the functional topology of the healthy brain and the ways it may be affected by disease or injury. This includes concepts of small-world organization, node centrality, modularity and assortative mixing^18^. Small-world organization is a particularly useful concept that has been well-studied in the neuroscience literature^19–24^. Small-world networks have the dual properties of dense local clustering among groups of nodes, but also short path lengths between all nodes in the brain^25^. These properties give rise to a system with efficient communication both at the local and the global scale, which is necessary for healthy, cost-efficient brain function^19,26^.

Previous studies have found evidence of changes in functional topology after moderate-to-severe TBI^27–32^, but examinations of concussion and mTBI have been more limited^33^. Studies of more severe TBI often report impaired small-world topology^32,34^, but little is known about the changes in small-world organization after concussion and how these changes evolve from acute injury to RTP and over the longer term. Even less is known about how small-world organization is related to clinical measures of outcome. To address these knowledge gaps, we acquired resting-state fMRI from a cohort of university-level athletes with concussion at acute injury, RTP and 1 year post-RTP. A large normative cohort of athletic controls without recent concussion was also imaged. We examined small-world measures of global and local efficiency over the recovery period and tested for the effects of clinical variables on network efficiency, including acute symptom severity and time to RTP. Based on TBI studies showing impaired small-world organization after TBI^32,34^, along with concussion studies showing connectivity changes lasting beyond RTP^35,36^, we hypothesized that concussed athletes would have reduced local and global network efficiency at acute injury, with effects that would not normalize until one year post-RTP. We further hypothesized that individuals with greater symptom burden and more prolonged recovery would have greater reductions in network efficiency at all post-concussion imaging sessions.

## Methods

### Study participants

Twenty-six (26) athletes recently diagnosed with a sport-related concussion were recruited from university-level sport teams at a single institution through the university sport medicine clinic (including volleyball, hockey, soccer, football, rugby, basketball, lacrosse and water polo; see Supplemental File 1 for athlete numbers by sport). Diagnosis was determined by a staff physician following direct or indirect contact to the head, with signs and/or symptoms as per the Concussion in Sport Group guidelines^37^. All concussed athletes completed imaging evaluations at the acute phase of injury (ACU), medical clearance to RTP (RTP) and one year post-RTP (1YR). The ACU imaging was conducted within 1 week of the concussion event (median and interquartile range : 4, [3 5] days). As a comparison group, one hundred and sixty-seven (167) athletic controls without recent concussion were also consecutively recruited and imaged at the start of their competitive season. The comparison group included balanced proportions of male and female athletes, without and with a history of concussion. Controls with a history of concussion were included, to ensure that the sample was representative of normal demographic variability and that it was an appropriate normative reference for concussed athletes, given that a large proportion of concussed athletes also had a history of concussion.

All athletes completed baseline assessments with the Sport Concussion Assessment Tool (SCAT)^38,39^ before the beginning of their respective athletic seasons, and athletes with concussion repeated the SCAT assessments at ACU and RTP. Athlete recruitment and data collection were carried out between October 2014 and March 2019. None of the athletes in this study had a history of neurological or psychiatric diseases or sensory/motor impairments. This study was carried out in accordance with recommendations of the Canadian Tri-Council Policy Statement 2 (TCPS2) and with approval of the research ethics boards at the University of Toronto and St. Michael’s Hospital, with written informed consent given by all subjects in accordance with the Declaration of Helsinki.

### Magnetic resonance imaging

Athletes were imaged at St. Michael’s Hospital using a research-dedicated MRI system operating at 3 T (Magnetom Skyra, Siemens, Erlangen, Germany) with the standard 20-channel head receiver coil, using the same sequences as reported in prior publications^36,40,41^. Structural imaging included 3D T1-weighted Magnetization Prepared Rapid Acquisition Gradient Echo (MPRAGE: inversion time (TI)/echo time (TE)/repetition time (TR) = 1090/3.55/2300 ms, flip angle (θ) = 8°, 192 sagittal slices with 240 × 240 mm field of view (FOV), 256 × 256 matrix, 0.9 mm slice thickness, 0.9 × 0.9 mm in-plane, 200 Hertz per pixel (Hz/px) bandwidth (BW)), fluid attenuated inversion recovery imaging (FLAIR: TI/TE/TR = 1800/387/5000 ms, 160 sagittal slices, 230 × 230 mm FOV with 512 × 512 matrix, 0.9 mm slice thickness, 0.4 × 0.4 mm in-plane, 751 Hz/px BW) and susceptibility-weighted imaging (SWI: TE/TR = 20/28 ms, θ = 15°, 112 axial slices with 193 × 220 mm FOV, 336 × 384 matrix, 1.2 mm slice thickness, 0.6 × 0.6 mm in-plane, 120 Hz/px BW). Structural images were reviewed in a 2-step procedure, with initial inspection by an MRI technologist and later review by a neuroradiologist, with clinical reporting if abnormalities were identified. No abnormalities (white matter hyper-intensities, contusions, micro-hemorrhage) were found for athletes in this study.

Resting-state fMRI was acquired via multi-slice T2*-weighted echo planar imaging (EPI: TE/TR = 30/2000 ms, θ = 70°, 32 oblique-axial slices with 200 × 200 mm FOV, 64 × 64 matrix, 4.0 mm slice thickness with 0.5 mm gap, 3.125 × 3.125 mm in-plane, 2298 Hz/px BW), producing a series of 195 brain volumes (6:30 min in total). During acquisition, athletes were instructed to lie still with their eyes closed and to not focus on anything. Data processing was performed as described in prior publications^36,40,41^ and was based on Analysis of Functional Neuroimages (AFNI; afni.nimh.nih.gov), the FMRIB Software Library (FSL; https://fsl.fmrib.ox.ac.uk) and customized algorithms. After discarding the first 4 images in each time series to allow magnetization to attain equilibrium, we performed rigid-body motion correction (AFNI *3dvolreg*), removal and interpolation of outlier volumes with SPIKECOR (nitrc.org/projects/spikecor), slice-timing correction (AFNI *3dTshift*), spatial smoothing with a 6 mm Full Width at Half Maximum (FWHM) isotropic 3D Gaussian kernel (AFNI *3dmerge*), along with regression of motion parameters and linear-quadratic trends. For motion parameter regression, principal component analysis was performed on the six rigid-body parameters, and the first two components were used as regressors. To control for physiological noise, the PHYCAA + algorithm (nitrc.org/projects/phycaa_plus) was used to down-weight non-neural signal, which was followed by regression of signal from white matter (WM) and cerebrospinal fluid (CSF). The WM and CSF regressions were performed following spatial normalization, described below.

The fMRI data were then co-registered to the common MNI152 anatomical template space. For each athlete, this was achieved using FSL *flirt* to estimate the rigid-body transform of their mean fMRI image to their T1-weighted scan, along with the 12-parameter affine transform of their T1 image to the MNI152 template. The affine matrices were then concatenated via FSL *convert_xfm* and the net transform was used to warp fMRI images to the MNI152 template, with resampling at 3 mm isotropic voxel resolution. To remove WM and CSF signal, subject T1-weighted images were segmented into grey matter (GM), WM and CSF maps using FSL *fast* and co-registered to the MNI152 template using the FSL *fslvbm* protocol, resampled into 3 mm isotropic voxel resolution. The maps were then smoothed using a 6 mm FWHM isotropic 3D Gaussian kernel and averaged across subjects, producing symmetric, cohort-specific probabilistic tissue templates. For WM, the mask of voxels within the 95th percentile of $p(WM)$ values was obtained and a single spatial erosion was performed (3 × 3 in-plane kernel). Two seed time series were obtained by separately averaging voxels within cerebral white matter and within brainstem white matter, as their time courses were substantially different. For CSF, the mask of voxels within the 95th percentile of $p(CSF)$ values was obtained. Two seed time series were then obtained by separately averaging voxels within the left and right lateral ventricles. The four physiological time series were afterwards regressed from each voxel, for each subject.

### Graph theoretic analysis

Functional networks were constructed by z-scoring the BOLD timeseries vectors ${{\varvec{x}}}_{v}$ of each dataset (where *v* = 1…68,500 indexes over voxels) and parcellating using the Brainnetome Atlas (BNA), which is a connectivity-based atlas that subdivides the brain into 246 cortical and subcortical regions ^42^. To account for cohort-specific variations in local topology and minor alignment errors that may affect the accuracy of parcellations, parcel time series ${{\varvec{s}}}_{k}$ (where *k* = 1…*246* indexes over parcels) were calculated as weighted averages of the voxels in each parcel. The voxel weights ${w}_{kv}$ were chosen to produce time series ${{\varvec{s}}}_{k}$ that explain the most variance per parcel, estimated at the group level. This was achieved by maximum likelihood fitting of a Gaussian mixture model of type #2 as in^43^, with fixed cluster assignments derived from the BNA. To reduce computational burden, the fitting was conducted based on a subsample of 34/167 (20%) controls that were demographically comparable to the full control group (mean age 20.3 ± 1.9 yrs., 17/34 (51%) female, 16/34 (51%) with history of concussion). For each subject and imaging session, we then calculated the set of parcel time series ${{\varvec{s}}}_{k}$ using the equation:

1 $${{\varvec{s}}}_{k}=\frac{1}{{\sum }_{v\in {z}_{k}}{w}_{kv}}{\sum }_{v \in {z}_{k}}{{\varvec{x}}}_{v}\bullet {w}_{kv}$$

Each ${{\varvec{s}}}_{k}$ is thus a weighted sum of BOLD time series ${{\varvec{x}}}_{v}$ assigned to the *k*th parcel, denoted ${z}_{k}$. We afterwards calculated the $246\times 246$ matrix of Pearson correlations between the parcel time series ${{\varvec{s}}}_{k}$, providing the functional networks for further analysis.

We afterwards examined graph-theoretic measures of small-worldness, calculated on thresholded functional networks ***G***, where each parcel represents a “node” and each supra-threshold connection represents an “edge” (i.e., binary undirected adjacency matrices). To ensure that effects were not driven by global differences in connectivity strength, metrics were evaluated at a percentile threshold *P* (i.e., all connections above the *P*th percentile = 1 and the rest = 0), calculated after excluding negative connections, since their interpretation is ambiguous. As graph-theoretic measures are sensitive to threshold choice, we explored *P* thresholds ranging from 1 to 50%, in increments of 1%. All graph-theoretic measures in this paper were calculated using the Brain Connectivity Toolbox (BCT; https://sites.google.com/site/bctnet/).

### Network efficiency

Small-worldness of the functional networks was characterized in terms of global efficiency (*E*_glob_) and local efficiency (*E*_loc_) of the adjacency matrices ***G***. This provides a general framework which is based on a single metric and computation does not require that graphs be fully connected^44^. For node pair *k* and *k*’, communication efficiency $e\left(k,k^{\prime}\right)$ is defined as the reciprocal of the shortest path length $d\left(k,k^{\prime}\right)$, i.e., the minimum number of edges that must be traversed to go from one node to the other:

2 $$e\left(k,k{^{\prime}}\right)=\frac{1}{d\left(k,k{^{\prime}}\right)}$$

For graph ***G*** consisting of *K* nodes, the average efficiency is defined as:

3 $$E\left({\varvec{G}}\right)=\frac{1}{K\left(K-1\right)}\sum_{k\ne k{^{\prime}}}e\left(k,k{^{\prime}}\right)$$

Computed over ***G***, this is its global efficiency ${E}_{glob}=E({\varvec{G}})$. We may also restrict our definition of efficiency to a local subgraph ***G***_k_, consisting of all neighbours of node *k*. The local efficiency of ***G*** is the average efficiency of all nodal subgraphs: ${E}_{loc}=\frac{1}{K}\sum E({{\varvec{G}}}_{k})$. Additionally, node-specific values of efficiency can be inspected, defined here as ${E}_{node}(k)=E({{\varvec{G}}}_{k})$, reflecting local resilience and connectedness of node *k* with respect to the rest of the brain. To simplify notation, the nodal index (*k*) will be omitted from future references to *E*_*node*_ but will remain implicit when discussing this metric.

**Figure 1:**
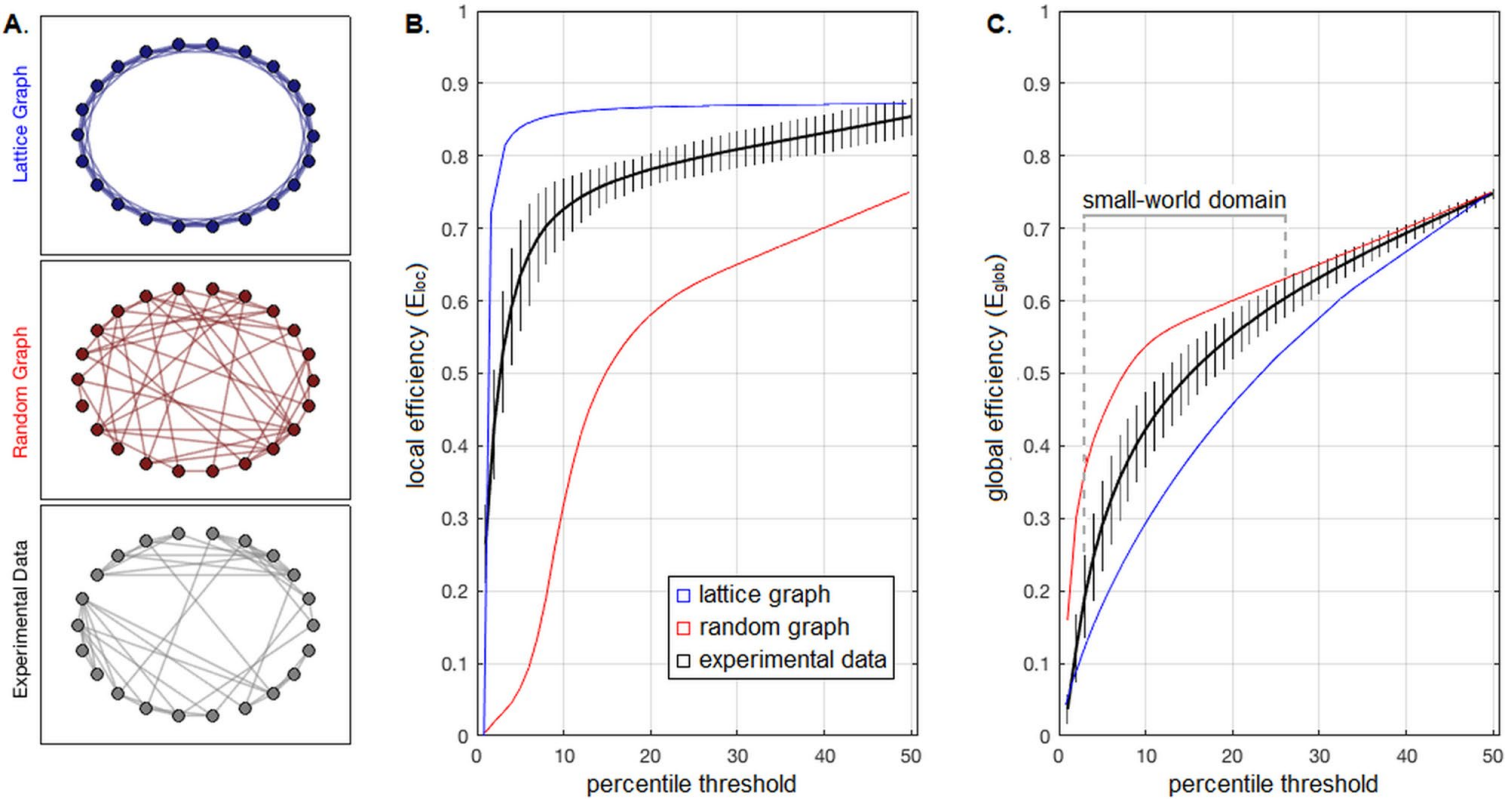
Characterizing network efficiency and small-world behaviour among healthy controls. (**A**) Examples of lattice and random graphs, along with a real-world graph of the experimental data from controls, plotted in circular layout. The latter graph is calculated from the average z-transformed connectivity matrix of BNA parcels in the occipital cortex (22 nodes), thresholded at *P* = 25% sparsity, with the former graphs generated at equivalent sizes and sparsity levels. (**B**,**C**) average local efficiency (*E*_loc_) and global efficiency (*E*_glob_) curves for healthy controls (black curve) along with sample 95% interval error bars, as a function of sparsity percentile threshold. The corresponding curves for lattice (blue) and random (red) graphs are also shown. The “small-world” domain is the interval in which 95% intervals do not overlap with either lattice or random graph efficiency curves for both efficiency measures (*P* = 3–26%).

To visualize how ${E}_{loc}$ and ${E}_{glob}$ measure small-world behaviour, consider the extreme case of a “lattice” graph, where each node is connected only to its k-nearest neighbours; an example is plotted in Fig. 1A (top panel). Here, neighbouring nodes are densely connected, facilitating local information flow, with short $d\left(k,k^{\prime}\right)$ in nodal subgraphs and correspondingly high *E*_loc_. However, distant nodes require many steps to reach, resulting in inefficient long-distance information flow, with long $d\left(k,k^{\prime}\right)$ in the full graph and therefore low *E*_glob_. We may then imagine randomly rewiring each connection until a fully random graph is obtained, as in Fig. 1A (middle panel). Now, distant nodes in the full graph can be reached in fewer steps, giving shorter $d\left(k,k^{\prime}\right)$ in the full graph and high ${E}_{glob}$. But this is at the expense of reducing connection densities between neighbouring nodes, with increased $d\left(k,k^{\prime}\right)$ in nodal subgraphs and low ${E}_{loc}$. Small-world networks are intermediate between these extremes, having densely connected groups of nodes with a small number of long-range connections between clusters. This is seen for sample experimental data in Fig. 1A (bottom panel), where we constructed a graph network from control data, consisting of all parcels in the occipital cortex. These networks have high *E*_glob_ and high *E*_loc_, reflecting efficient information flow at global and local scales.

To avoid issues of threshold selection for the binary graphs, we identified the regime ${P}_{min}\le P\le {P}_{max}$ where networks had small-world behaviour, with *E*_glob_ and *E*_loc_ values intermediate between lattice and random graphs^22^. We then obtained the integrated efficiency:

4 $$IE={\sum }_{P={P}_{min}}^{{P}_{max}}E(P)\Delta P$$

This approach, which has been used in prior literature^21,22,45,46^, produced *IE*_glob_, *IE*_loc_ and *IE*_node_ values, which were used to study concussion effects. Prior to integration, to control for distribution tails that vary in size by threshold, and to improve estimation robustness, efficiency values were winsorized at the 95^th^ percentile (2-tailed) over all subjects, concussed and control.

### Analysis of demographic and clinical data

The demographics of concussed athletes and controls are reported in Table 1, including age, sex and prior concussion history, along with time to RTP for concussed athletes, defined as the number of days from concussion to symptom resolution following a graded exertion protocol^4^. From the SCAT, a symptom severity score was obtained by summing across the 22-item scale, with each item receiving a 7-point Likert scale rating. A total symptoms score was also obtained by counting symptoms with non-zero ratings. Brief cognitive testing scores were also reported, including Orientation, Immediate Memory, Concentration and Delayed Memory, along with total scores for the modified balance error scoring system (M-BESS). All scores were tested at acute injury and RTP for a significant difference relative to baseline, via non-parametric Wilcoxon paired-measures tests. As per evolving clinical guidelines, all concussed athletes and 68/167 controls were evaluated with SCAT3, while the remainder were evaluated using SCAT5. Immediate Memory and Delayed Memory tests had changed in SCAT5 (from 15 to 30 items and from 5 to 10 items, respectively). For these subtests, statistics were only reported for SCAT3 data, as this was collected for all concussed athletes. For other subtests, statistics were based on the complete dataset, after confirming there were no within-cohort differences in SCAT3 and SCAT5 scores based on 2-sample Wilcoxon tests (*p* ≥ 0.288, for all measures).

**Table 1:** Demographic and clinical data for athletes with concussion and controls. All non-binary variables except age are summarized by the median and interquartile range [Q1, Q3].

	Control	Concussion		
Age (mean ± SD)	20.2 ± 2.0	19.9 ± 1.9		
Female	87/167 (52%)	14/26 (54%)		
Prior concussion	73/167 (44%)	14/26 (54%)		
Number of prior concussions	2 [1, 2]	2 [1, 3]		
Months since prior concussion	32 [12, 49]	26 [4, 60]		
Days to RTP	–	28 [15, 55]		

	Baseline	Baseline	Acute	RTP
Total symptoms	2 [0, 5]	4 [2, 8]	10 [5, 17]*	0 [0, 2]
Symptom Severity	3 [0, 7]	6 [2, 13]	18 [6, 32]*	0 [0, 2]
Orientation	5 [5]	5 [5]	5 [5]	5 [5]
Immediate memory^+^	15 [14, 15]	14 [14, 15]	15 [14, 15]	15 [14, 15]
Concentration	4 [3, 4]	4 [3, 4]	4 [3, 5]	4 [3, 5]
Delayed memory^+^	4 [3, 5]	5 [3, 5]	4 [3, 5]	5 [4, 5]
M-BESS errors	1 [0, 3]	3 [1, 4]	3 [0, 5]	1 [0, 2]

For tests of Immediate Memory and Delayed Memory, denoted by a ‘^+^’, statistics are based on a reduced sample of 68/167 controls, due to changes in scoring guidelines between SCAT3 and SCAT5. Significant differences in clinical scores at acute injury, relative to athletes' own baseline, are noted with a ‘*’.

### Analysis of network efficiency for healthy controls

To identify the regime of small-world behaviour, we plotted average whole-brain ${E}_{loc}$ and ${E}_{glob}$ as a function of threshold *P* for healthy controls, with sample 95% intervals. For comparison purposes, the curves were also plotted for lattice and random graphs. The domain of small-world behaviour was identified as the range of thresholds where the 95% intervals of experimental ${E}_{loc}$ and ${E}_{glob}$ values did not overlap lattice or random curves. From this interval, we calculated ${IE}_{loc}$ and ${IE}_{glob}$, along with nodal values ${IE}_{node}$. Additional analyses tested for effects of demographics on ${IE}_{loc}$ and ${IE}_{glob}$. Efficiency values were regressed onto covariates of age (integer), sex (binary) and history of concussion (binary), using a bootstrapped general linear model (GLM; 1000 resamples) to obtain regression coefficients $b$, with 95% confidence intervals (95% CIs), bootstrap ratios (BSRs; z-distributed estimates of standardized effect size, calculated as $b$ divided by standard error) and empirical p-values based on the fraction of resamples overlapping zero. Similar analyses were conducted among controls with a history of concussion, for number of prior concussions and months since last injury (integer values). Significant variables were then identified at a false discovery rate (FDR) threshold of 0.05.

### Analysis of network efficiency for concussed athletes

For concussed athletes, we compared ${IE}_{loc}$ and ${IE}_{glob}$ at each imaging session (ACU, RTP, 1YR) to the control group values, with 2-sample bootstrap analysis (1000 resamples) used to obtain mean differences, 95%CIs, BSRs and p-values. Similarly, we measured longitudinal change in ${IE}_{loc}$ and ${IE}_{glob}$ of concussed athletes between imaging sessions, with paired-measures bootstrap analysis (1000 resamples) used to obtain mean change, 95%CIs, BSRs and p-values. Significant effects were identified after adjusting over time points at an FDR of 0.05. Subsequent analyses tested for effects of clinical variables on ${IE}_{loc}$ and ${IE}_{glob}$. Covariates included total symptom severity at acute injury and days to RTP. Given the high correlation between these variables (see “Results” below), a pair of orthogonal composite scores (CS) were defined. After the two variables were renormalized via inverse empirical distribution function and mean centered, composite score one (CS1) was computed as the sum of variables (acute symptoms + days to RTP), which quantified overall severity of clinical outcome. Composite score two (CS2) was computed as the difference (acute symptoms− days to RTP), which quantified discrepancy between the measures of concussion outcome (i.e., a positive score denotes high symptom burden but rapid recovery, and a negative score denotes the converse). The CS effects were evaluated using a bootstrapped GLM (1000 resamples) at each imaging session to obtain $b$ with 95%CIs, BSRs and p-values and significant effects were identified at an FDR of 0.05.

### Analysis of nodal efficiency measures

The previous sections focused on whole-brain topological measures of efficiency, with *IE*_loc_ being used to compare the mean nodal efficiency of concussed and controls groups. In this section, we also tested for concussion-related changes in the distribution of nodal efficiency values about the mean. The set of average control *IE*_node_ values were plotted against average concussed *IE*_node_ values for each imaging session, and the regression line of best fit obtained with slope coefficient β. A flatter slope (β < 1) indicated reduced variability of nodal values about the mean, whereas a steeper slope (β > 1) indicated increased variability about the mean. Bootstrap resampling of subjects was conducted (1000 resamples) to obtain 95%CIs and p-values, based on the fraction of resamples overlapping β = 1. We also visualized the spatial patterns of change in nodal efficiency. The mean *IE*_node_ map was plotted for controls and, for tests showing a global effect (i.e., β values differing from 1), 2-sample bootstrap analyses comparing concussed to controls were used to obtain BSRs, denoting the standardized effect sizes, thresholded at a nominal |BSR|>2 (approximately p < 0.05, uncorrected).

### Comparison with other graph theoretic indices

We also compared network efficiency with other graph-theoretic measures, to enhance our understanding of the effects of concussion on brain topology. Node degree (*DEG*) provides a simple measure of connectedness; it is obtained by counting the number of non-zero edges in ***G***, for a given node. Node centrality is used to assess the importance of individual nodes in overall network cohesion. There exist many different measures of centrality, including betweenness centrality (*CB*), which is obtained by counting the number of times a given node is included in the shortest-distance path between two other nodes^47^, and eigenvector centrality (*CE*), which is obtained from the first eigenvector of ***G*** and reflects the tendency of a node to be connected to other highly connected nodes^48^. Network modularity (*MOD*) evaluates the tendency of the network to organize into “modules” that have dense within-module connections but sparse between-module connections, which can be expressed as:

5 $$Q=\frac{1}{2m}{\sum }_{k,k{^{\prime}}}\left({\varvec{G}}(k,k{^{\prime}})-[DEG(k)*DEG({k}^{{^{\prime}}})]/2m\right)\delta (k,k{^{\prime}})$$

where *m* is the total number of edges in the graph, ***G***(*k*, *k*’) is the connection between nodes *k* and *k*’, *DEG*(*k*) denotes the degree of the *k*^th^ node, and $\delta (k,k^{\prime})$ equals 1 if *k* = *k*’ and 0 otherwise^49^. In this paper, *MOD* is calculated on a fixed community structure, estimated at the group level using the Infomap algorithm (https://www.mapequation.org/code.html) and the BCT consensus clustering algorithm (see Supplemental File 2 for details). Lastly, degree assortativity (*DAS*) measures the tendency for nodes to connect to nodes of similar degree^50^; it is calculated as the correlation of each node’s degree with the average degree of its neighbours.

For consistency with the efficiency measures, the other graph-theoretic measures were also integrated over the small-world regime of ${P}_{min}\le P\le {P}_{max}$ as described in the *Network Efficiency* section above. We first characterized the relationships between graph measures in healthy controls, by computing the Spearman correlations of *IE*_loc_ and *IE*_glob_ values with other global network indices, along with the bootstrapped 95%CIs. This included centrality values averaged over all nodes, denoted *ICB*_avg_ and *ICE*_avg_, along with *IMOD* and *IDAS*. Note that *IDEG*_avg_ was not included, as this has a fixed value at a given percentile threshold. We also compared concussed athlete values at each imaging session (ACU, RTP, 1YR) to the control group and tested for longitudinal changes in concussed athlete values, using the bootstrapping procedures described in the *Analysis of network efficiency for concussed athletes* section above. Mean nodal maps were also plotted for *IDEG*_node_, *ICB*_node_ and *ICE*_node_, and reliable changes in nodal efficiency were assessed using bootstrap analysis, as described in *Analysis of regional efficiency measures* above, with results included in Supplemental File 4.

## Results

Demographic and clinical data are summarized in Table 1 for the control and concussed groups. Both cohorts included a balanced sample of male and female athletes, with and without history of concussion. Both groups had comparable proportions with prior concussion, and injury characteristics were comparable, including number of prior concussions (z = − 1.28, p = 0.200) and time since last injury (z = 0.63, p = 0.531), reinforcing that these cohorts are demographically similar. For the concussed athletes, symptoms were significantly elevated at acute injury relative to baseline and controls, in terms of number of symptoms and symptom severity (p < 0.001, for all tests); but neither was significantly elevated at RTP (p ≥ 0.99, for all tests). Symptom severity and time to RTP were highly variable between concussed athletes, however, and the two variables have a relatively high Spearman correlation (ρ = 0.668; 95%CI: [0.402 to 0.823]; *p* < 0.001). None of the concussed athletes had sustained a new concussion between acute injury and one year post-RTP, and all athletes had returned to normal school/work, social and sport activities at this time, based on clinic follow-up.

Figure 1 depicts small-world behaviour of the healthy controls, with Fig. 1A showing examples of simulated lattice and random graphs, along with a small-world graph of similar size obtained from experimental data. For whole-brain networks, Fig. 1B shows that *E*_loc_ is consistently intermediate between the extreme lattice and random graphs, for nearly the full range of tested thresholds. Conversely, *E*_glob_ is more restricted, with 95% sampling intervals overlapping “lattice” graphs at high levels of sparsity *P* < 3% and overlapping “random” graphs at low levels of sparsity *P* > 26%. Thus, we chose the interval of *P* = 3–26% for analysis of integrated efficiency measures. Subsequent analysis of demographics identified modest negative associations between *IE*_glob_ and age (*b* and 95% CI:  − 0.040, [− 0.072, − 0.003], BSR = − 2.20, *p* = 0.039) and weaker effects otherwise (|BSR|≤ 1.62 and *p* ≥ 0.122, for all other tests), none of which were significant at an FDR threshold of 0.05. Among the controls with a history of concussion, the efficiency measures had non-significant associations with the number of prior concussions and number of months since last concussion (|BSR|≤ 1.64 and *p* ≥ 0.071, for all tests).

**Figure 2:**
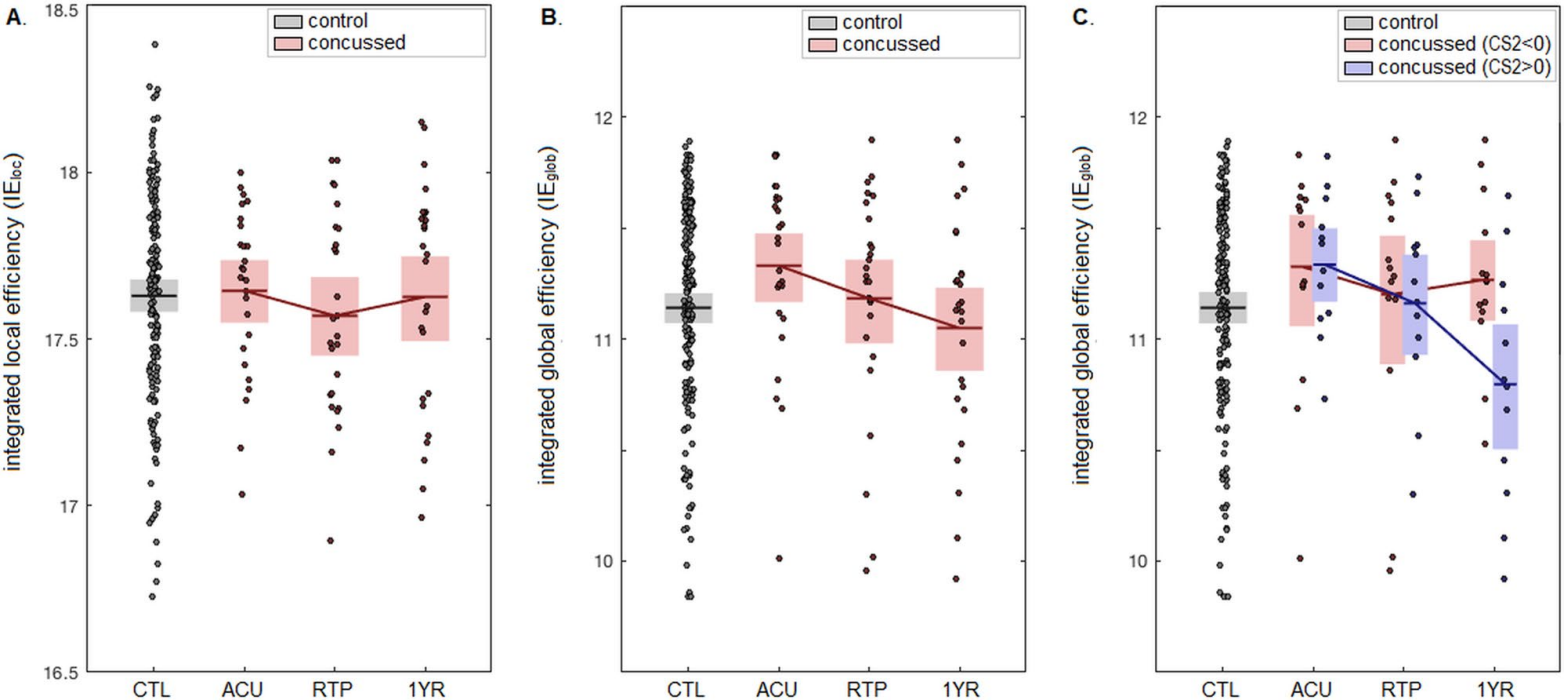
Effects of concussion on network efficiency. (**A**,**B**) depict the integrated local efficiency (*IE*_loc_) and integrated global efficiency (*IE*_glob_) values for healthy controls (black) and for concussed athletes (red) at each imaging session. (**C**) depicts the *IE*_glob_ values for controls and concussed athletes, subgrouped based on clinical score CS2 (days to RTP symptom severity). Horizontal bars represent sample means and shaded boxes denote the 95%CIs of the mean.

The effects of concussion on measures of *IE*_loc_ and *IE*_glob_ are shown in Fig. 2, with Table 2 summarizing cross-sectional and longitudinal effects. As seen in Fig. 2A, the *IE*_loc_ values of concussed athletes are comparable to controls at all imaging sessions, with no significant longitudinal changes between sessions. As shown in Fig. 2B, the *IE*_glob_ values of concussed athletes are elevated relative to controls at ACU, becoming more comparable at later imaging sessions, but with non-significant differences at an FDR of 0.05. The *IE*_glob_ values show statistically significant longitudinal declines, though, with both RTP and 1YR sessions being lower than ACU. Supplemental cross-sectional analyses adjusting for demographic covariates of age, sex and concussion history in a bootstrapped GLM framework found no significant impact on the parameter estimates (BSR ≤ 0.81, p ≥ 0.350, for all tests).

**Table 2:** Effects of concussion on integrated local efficiency (*IE*_loc_) and integrated global efficiency (*IE*_glob_), for concussed athletes at acute injury (ACU), return to play (RTP) and one year post-RTP (1YR). This includes cross-sectional differences relative to controls (CTL) and within-subject longitudinal changes between imaging sessions. Tables report mean effects with bootstrapped 95% confidence intervals (95% CIs), bootstrap ratios (BSRs) and p-values. Significant effects at an FDR threshold of 0.05 are noted with ‘*’.

		IE_loc_				IE_glob_			
		Mean	95%CI	BSR	p	Mean	95%CI	BSR	p
Cross-sectional	ACU-CTL	0.015	[− 0.090, 0.119]	0.28	0.739	0.190	[0.008, 0.352]	2.14	0.032
	RTP-CTL	− 0.060	[− 0.182, 0.071]	− 0.93	0.351	0.042	[− 0.170, 0.234]	0.41	0.657
	1YR-CTL	− 0.003	[− 0.137, 0.129]	− 0.04	0.932	− 0.091	[− 0.287, 0.113]	− 0.87	0.342
Longitudinal	RTP-ACU	− 0.075	[− 0.166, 0.020]	− 1.57	0.129	− 0.147	[− 0.295, − 0.004]	− 2.01	0.039*
	1YR-ACU	− 0.018	[− 0.164, 0.121]	− 0.26	0.790	− 0.281	[− 0.514, − 0.057]	− 2.41	0.011*
	1YR-RTP	0.057	[− 0.0714, 0.182]	0.88	0.346	− 0.133	[− 0.360, 0.088]	− 1.17	0.237
CS1 regression	ACU	0.037	[− 0.474, 0.674]	0.13	0.888	0.873	[− 0.017, 1.948]	1.76	0.044
	RTP	0.287	[− 0.377, 1.063]	0.81	0.384	0.657	[− 0.476, 1.874]	1.11	0.237
	1YR	− 0.484	[− 1.144, 0.255]	− 1.36	0.197	− 0.299	[− 1.331, 0.907]	− 0.53	0.572
CS2 regression	ACU	− 0.121	[− 1.271, 1.151]	− 0.20	0.851	− 0.405	[− 2.074, 1.605]	− 0.43	0.657
	RTP	− 0.522	[− 1.520, 0.846]	− 0.87	0.399	− 0.081	[− 2.481, 2.127]	− 0.07	0.934
	1YR	− 0.586	[− 2.079, 1.071]	− 0.73	0.519	− 2.844	[− 5.052, − 0.738]	− 2.59	0.004*

The effects of clinical scores on *IE*_loc_ and *IE*_glob_ were also evaluated, with Table 2 summarizing covariate effects. The clinical score CS1, reflecting overall clinical severity, had a modest positive association with *IE*_glob_ but showed weaker effects otherwise, and none of the CS1 analyses were significant at an FDR of 0.05. Conversely, clinical score CS2, reflecting atypicality of recovery, showed a significant negative association with *IE*_glob_ at one year post-RTP, although effects were otherwise substantially weaker. As shown in Fig. 2C, the negative relationship of CS2 with *IE*_glob_ at 1YR indicates that athletes with CS2 < 0 (i.e., relatively high symptoms but rapid recovery) had normalized to match control values by one year post-RTP (mean and 95% CI:  − 0.126, [− 0.073, − 0.322], BSR = 1.22, *p* = 0.220), whereas athletes with CS2 > 0 (i.e., relatively low symptoms but more prolonged recovery) had reductions in network *IE*_glob_ which were significantly lower than controls at this time (− 0.344, [− 0.637, − 0.044], BSR = − 2.23, *p* = 0.022).

**Figure 3:**
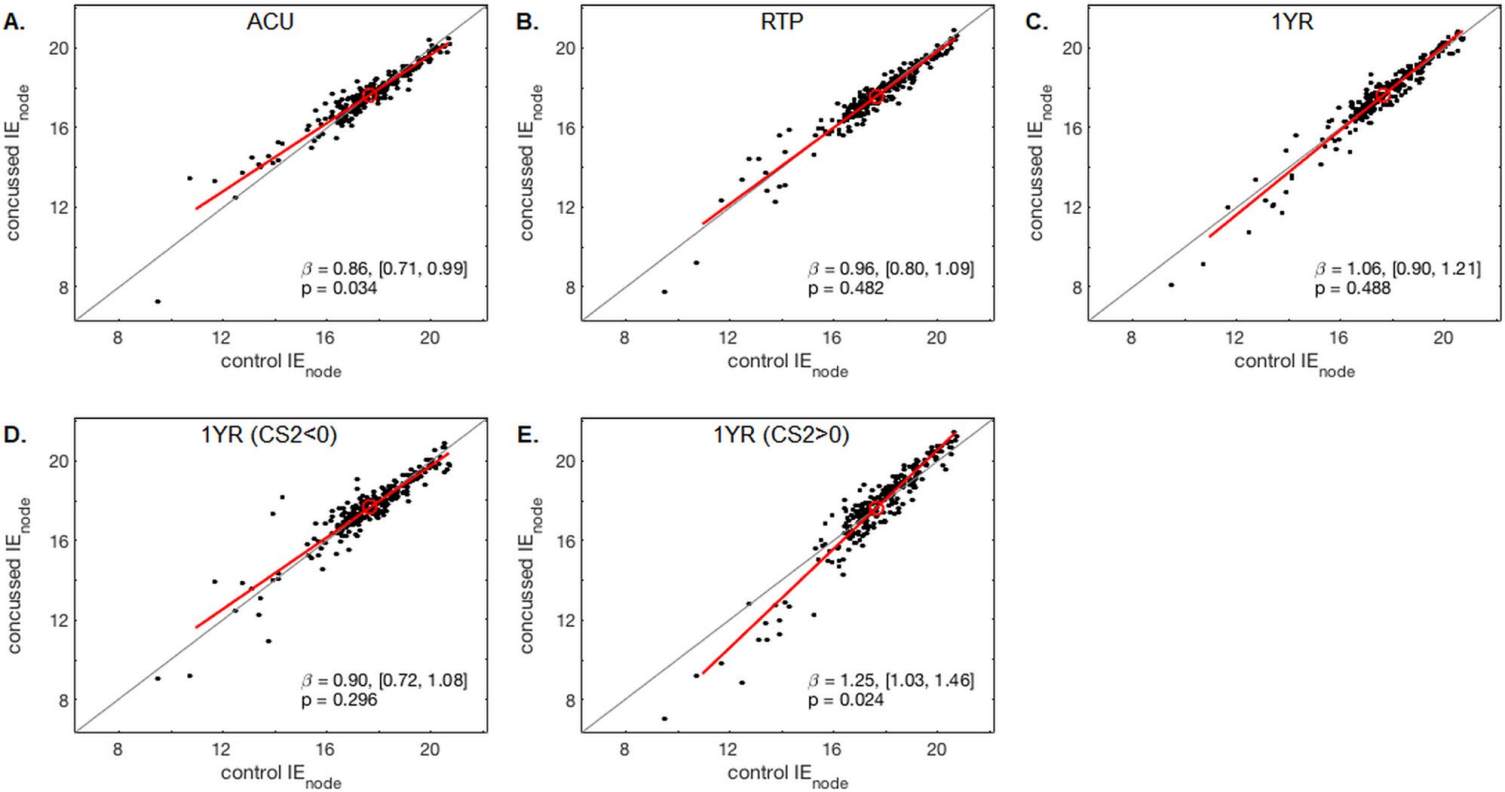
Effects of concussion on nodal efficiency distributions. Points in the scatterplots represent mean integrated nodal efficiency (*IE*_node_) values of concussed athletes, for a given node, plotted against the corresponding mean of athletic controls. The scatterplots are shown for (**A**) acute injury, (**B**) return to play (RTP) and (**C**) 1 year post-RTP. Additional scatterplots show mean *IE*_node_ values of the concussed athletes at one year post-RTP, subgrouped on clinical score CS2 (days to RTP symptom severity), with (**D**) CS2 < 0 and (**E**) CS2 > 0. For all scatterplots, the line of best fit is given (red line) along with the nodal mean (red circle); the line of identity is also plotted (grey line). The slope coefficient β is also given, along with bootstrapped 95%CIs and empirical p-values.

Figure 3 provides a more detailed investigation of nodal efficiency, by plotting mean *IE*_node_ values of controls against the corresponding values of concussed athletes. For ACU, the scatterplot slope β is consistently less than unity, indicating a systematic “shift” of individual *IE*_node_ values towards the nodal mean *IE*_loc_, where the highest nodal values for concussed athletes are most decreased and the lowest nodal values are most increased, i.e., *IE*_node_ values were more uniform among concussed athletes compared to controls. At RTP and 1YR, there is a gradual increase in β, but the slopes do not consistently deviate from unity, indicating that the distributions of mean nodal values are comparable to controls. Dividing the concussed athletes into subgroups of CS2 < 0 and CS2 > 0 at 1YR, the athletes with CS2 < 0 had a β that did not differ substantially from controls. However, athletes with CS2 > 0 had a β that was consistently greater than unity, indicating greater dispersion of *IE*_node_ values about the nodal mean *IE*_loc_, where the highest nodal values were most increased and the lowest nodal values were most decreased.

**Figure 4:**
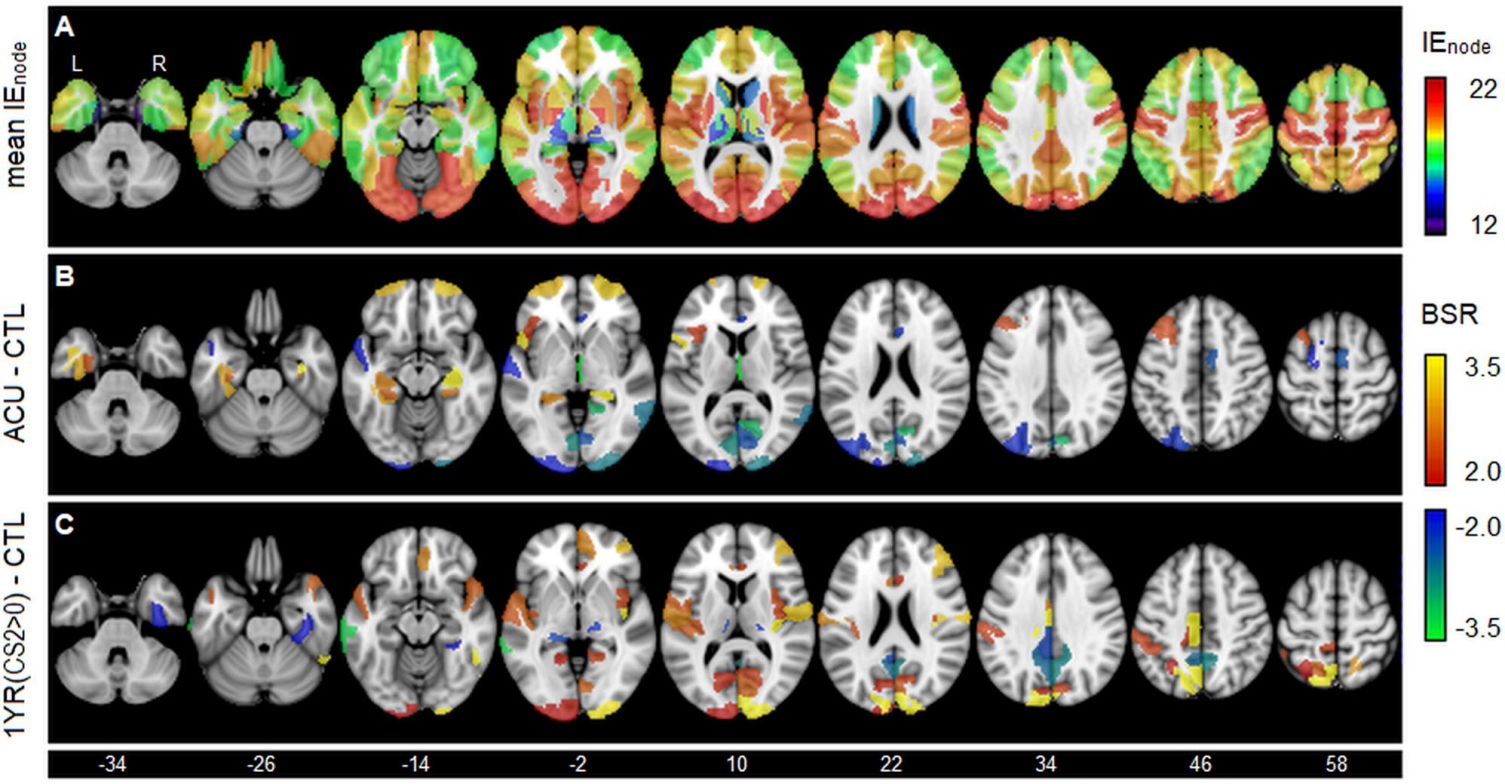
Localizing the effects of concussion on nodal efficiency. (**A**) The mean integrated nodal efficiency (*IE*_node_) values, averaged over all controls. Standardized effects sizes, reported as bootstrap ratio (BSR) values, are shown for contrasts of (**B**) concussed athletes at acute injury (ACU) relative to controls (CTL), and for (**C**) concussed athletes with clinical score CS2 > 0 at one year post-RTP (1YR) relative to controls (CTL). Maps are thresholded at |BSR|> 2, equivalent to *p* < 0.05 uncorrected, and the z-axis coordinates of axial slices in MNI space are provided.

Figure 4 localizes areas of nodal efficiency difference relative to controls, for the analyses showing consistent differences from controls in Fig. 3. The mean map of *IE*_node_ in Fig. 4A shows highest values primarily in occipital, insular, precentral and postcentral regions, along with the cuneus and precuneus, indicating that nodes in these regions are all part of densely connected clusters. Intermediate values are found in prefrontal and parietal regions, where values decrease with greater laterality, while the lowest values are seen subcortically. As shown in Fig. 4B and summarized in Supplemental File 3, concussed athletes at ACU have reduced *IE*_node_ mainly in posterior occipital areas of higher efficiency and increased *IE*_node_ mainly in frontal and subcortical areas of lower efficiency, with a balanced proportion of regions showing increases and decreases. In comparison, as shown in Fig. 4C and summarized in Supplemental File 3, the concussed athlete subgroup with CS2 > 0 at 1YR shows an opposite pattern of effect, with increased *IE*_node_ posteriorly, mainly within occipital and parietal areas. Negative effects were sparser and seen more in areas of intermediate nodal efficiency, including precuneal, middle temporal and posterior cingulate regions.

**Figure 5:**
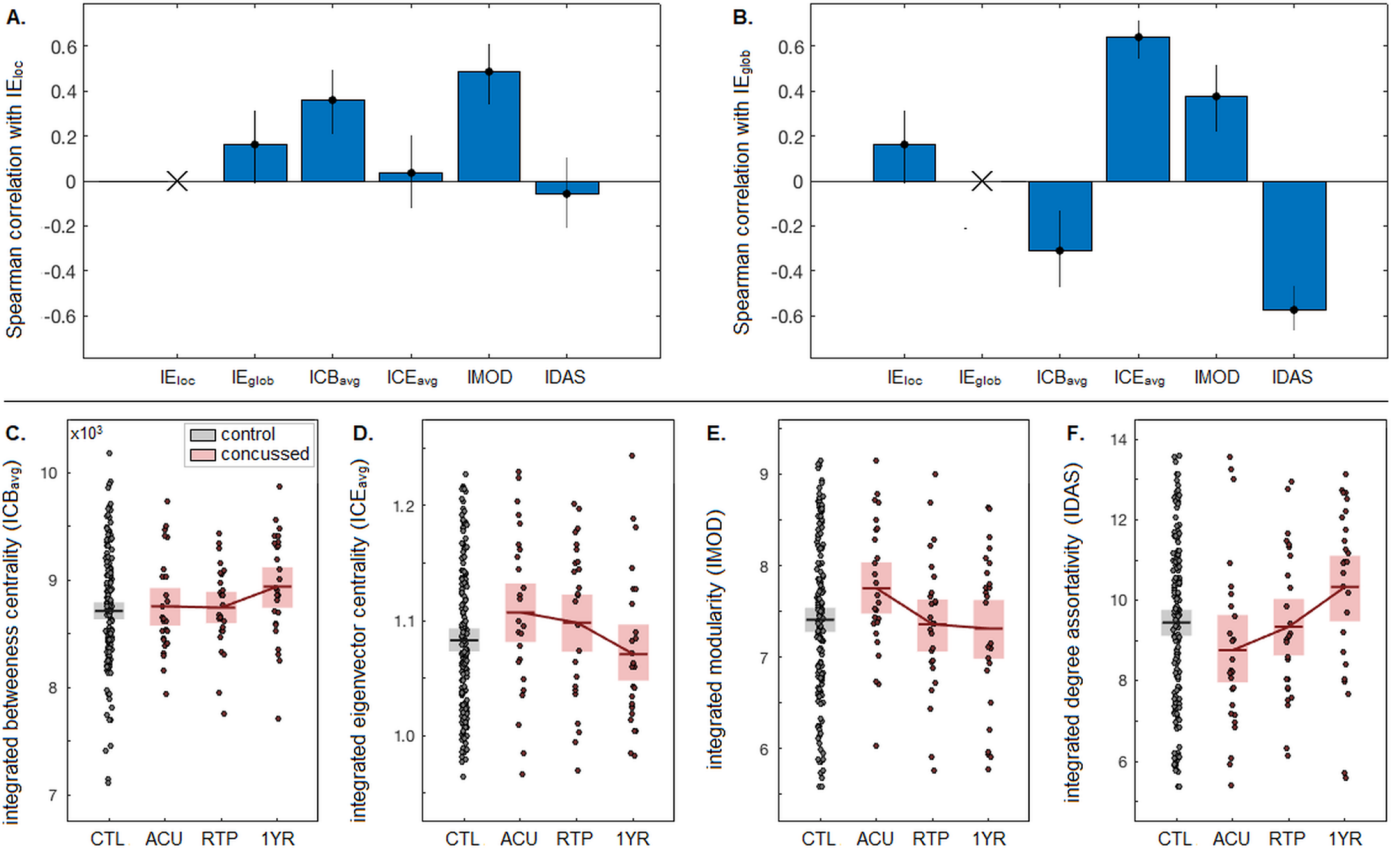
Relationship between network efficiency and other graph-theoretic measures. This includes Spearman correlations of healthy control values, for (**A**) integrated local efficiency (*IE*_loc_) and (**B**) integrated global efficiency (*IE*_glob_), with respect to integrated measures of mean betweenness centrality (*ICB*_avg_), mean eigenvector centrality (*ICE*_avg_), modularity (*IMOD*) and degree assortativity (*IDAS*). (**C**,**D**,**E**,**F**) depict *ICB*_avg_, *ICE*_avg_, *IMOD* and *IDAS* values for healthy controls (black) and for concussed athletes (red) at each imaging session. Horizontal bars represent sample means and shaded boxes denote the 95%CIs of the mean.

Figure 5 characterizes the relationships between network efficiency and other graph-theoretic measures. The correlations between measures for healthy controls are shown in Fig. 5A,B, with *IE*_loc_ and *IE*_glob_ having very weak positive correlations. The centrality measures show different relationships with network efficiency, as *ICB*_avg_ has a weak positive correlation with *IE*_loc_ but a negative correlation with *IE*_glob_. Conversely, *ICE*_avg_ shows no association with *IE*_loc_, but a moderate positive correlation with *IE*_glob_. The modularity index *IMOD* shows weak positive correlations with *IE*_loc_ and *IE*_glob_, whereas associativity index *IDAS* shows no associations with *IE*_loc_ but moderate negative correlations with *IE*_glob_.

The effects of concussion on the other graph-theoretic measures are shown in Fig. 5C–F, with standardized effect sizes and p-values reported in Table 3. The plots show increasing *ICB*_avg_ and *IDAS* values and decreasing *ICE*_avg_ and *IMOD* values during concussion recovery, although only *IDAS* and *IMOD* had significant longitudinal changes at an FDR threshold of 0.05. Deviations from the control cohort were generally limited, with *IMOD* showing modest elevations at ACU, and *ICB*_avg_ and *IDAS* showing modest elevations at 1YR, although none of the differences were statistically significant. Relationships with clinical scores were also limited, with CS1 having significant negative associations with *ICE*_avg_ and *IMOD* at 1YR and CS2 having a modest but non-significant association with *ICE*_avg_ at 1YR.

**Table 3:** Effects of concussion on integrated measures of mean betweenness centrality (*ICB*_avg_), mean eigenvector centrality (*ICE*_avg_), modularity (*IMOD*) and degree assortativity (*IDAS*), for concussed athletes at acute injury (ACU), return to play (RTP) and one year post-RTP (1YR). This includes cross-sectional differences relative to controls (CTL) and within-subject longitudinal changes between imaging sessions. Tables report bootstrap ratios (BSRs) and p-values. Significant effects at an FDR threshold of 0.05 are noted with ‘*’.

		*ICB*_avg_		*ICE*_avg_		*IMOD*		*IDAS*	
		BSR	p	BSR	p	BSR	p	BSR	p
Cross-sectional	ACU-CTL	0.44	0.718	1.64	0.108	2.22	0.029	− 1.57	0.111
	RTP-CTL	0.36	0.677	1.07	0.273	− 0.31	0.725	− 0.27	0.806
	1YR-CTL	2.23	0.040	− 0.88	0.367	− 0.54	0.561	2.01	0.044
Longitudinal	RTP-ACU	− 0.11	0.134	− 0.49	0.614	− 2.69	0.009*	1.15	0.254
	1YR-ACU	1.60	0.118	− 2.00	0.042	− 2.31	0.025*	2.78	0.010*
	1YR-RTP	1.56	0.938	− 1.63	0.100	− 0.24	0.805	2.05	0.034*
CS1 regression	ACU	− 0.31	0.721	0.19	0.816	0.84	0.382	− 0.83	0.372
	RTP	0.85	0.386	− 0.70	0.495	1.05	0.261	0.52	0.592
	1YR	− 2.55	0.013*	0.27	0.823	− 2.97	0.003*	− 0.21	0.857
CS2 regression	ACU	0.55	0.541	0.38	0.710	− 0.33	0.731	− 0.61	0.548
	RTP	0.87	0.378	1.24	0.226	0.14	0.939	− 1.21	0.224
	1YR	0.89	0.349	− 2.24	0.033	− 0.99	0.355	1.76	0.106

## Discussion

There is a growing body of literature examining how functional connectivity is altered after a concussion. To date, these studies have largely focused on changes in the strength of pairwise connections. Less is known about longitudinal changes in whole-brain topology, and whether there is evidence of persistent topological changes beyond medical clearance to RTP. The present study addressed this knowledge gap by imaging concussed athletes at acute injury, RTP and one year post-RTP, along with a substantial control cohort. Whole-brain functional topology was studied using measures of local and global efficiency *IE*_loc_ and *IE*_glob_ to quantify small-world behaviour^44^. This study found no significant post-concussion alterations in *IE*_loc_, whereas *IE*_glob_ was elevated at acute injury and had declined significantly by RTP. These findings are contrary to our primary hypothesis, which predicted reduced *IE*_loc_ and *IE*_glob_ at acute injury, with effects lasting beyond RTP. Concussed athletes with atypically long recovery, given their acute symptoms, also had significantly reduced *IE*_glob_ at 1 year post-RTP. These findings differ somewhat from our secondary hypothesis, which predicted that both symptom severity and prolonged recovery would correlate with reduced *IE*_loc_ and *IE*_glob_ at all sessions. Post-concussion changes in functional network efficiency may therefore occur that are distinct from more severe TBI, with effects lasting well beyond medical clearance to RTP for some individuals.

This study used a substantial sample of healthy controls to characterize small-world behaviour and provide a normative reference for concussed athletes. The controls had *E*_loc_ and *E*_glob_ values that were intermediate between lattice graphs (high *E*_loc_, low *E*_glob_) and random graphs (low *E*_loc_, high *E*_glob_) across a wide range of thresholds. Local efficiency was relatively insensitive to threshold choice, whereas global efficiency had a narrower band of thresholds in which small-world behaviour was seen, which is consistent with prior literature^22^. When examining the effects of demographics on network efficiency, there was a modest tendency towards reduced *IE*_glob_ in older athletes. The small effect may be due to the narrow age range under investigation, as a previous study found significantly reduced *IE*_loc_ and *IE*_glob_ in a group of older adults (mean age: 67 years) compared to a group of young adults (mean age: 25 years)^22^. It is also unclear if the age-related decrease in the present study is due to normal maturation or exposure to sub-concussive impacts over years of sport participation. The present study found sex effects on network efficiency to be negligible. This may be due to the focus on whole-brain measures, as a previous study reported more localized hemisphere- and node-specific sex differences in network efficiency^21^; a deeper investigation into sex effects is an important area of future research. History of concussion also showed negligible effects on network efficiency. For a previous study of moderate-to-severe TBI^27^, subjects who were scanned an average of 4–5 years post-injury had unimpaired small-world topology but had modest increases in *E*_loc_. This inconsistency may be due to differences in injury severity and may be further compounded by issues of accuracy in the retrospective reporting of concussion history.

When analyzing concussed athletes, our primary finding was elevated mean *IE*_glob_ at acute injury, which had significantly declined at RTP, whereas *IE*_loc_ showed no significant effects. The elevated *IE*_glob_ values at acute injury may be interpreted as evidence of shorter inter-nodal path lengths throughout the brain, thereby facilitating global information flow. This result is consistent with models that describe de-differentiation and hyper-integration as adaptive responses to mTBI^51^. It must be emphasized, however, that *IE*_glob_ deviations from controls at acute injury were modest and non-significant after adjusting for multiple comparisons, indicating that the mean effect of concussion on whole-brain network efficiency is subtle compared to more severe forms of TBI^32,34^. The absence of altered *IE*_loc_ values also indicates that concussion does not affect the tendency of brain regions organize into clusters, which is important, as this is required for a robust, fault-tolerant network^19^. Furthermore, the mean effects of concussion appear to be limited to the acute phase of injury, as *IE*_glob_ values are comparable to controls at RTP and show no longitudinal change afterwards. The present findings differ from earlier functional connectivity studies in this cohort, which found acute elevations in global connectivity strength that remained present at RTP^35,36^. The discrepancy suggests that persistent elevations in global connectivity reflect a “scaling” of connectivity between regions, without changes in the underlying topological organization. This is supported by a study of network topology in non-sport mTBI, where *IE*_loc_ and *IE*_glob_ values were comparable to controls at 4 weeks post-injury^46^.

For clinical covariates, we examined composite measure CS1 (acute symptom severity + days to RTP), which indexes the overall severity of outcome, and CS2 (days to RTP—acute symptom severity), which indexes the “atypicality” of outcome. No significant associations were found between CS1 and network efficiency after adjusting for multiple comparisons, although a modest positive association was seen between CS1 score and *IE*_glob_ at acute injury, indicating that heightened efficiency may be a subtle early indicator of more severe clinical outcome. These results also contrast with previous studies in this cohort, where greater symptom severity and CS1 scores were associated with reduced global connectivity strength^7,36^, further emphasizing the distinction between connectivity scaling and topological organization. A significant negative association was found between CS2 scores and *IE*_glob_ at one year post-RTP. This suggests that, for individuals where recovery time is disproportionately long given their acute symptoms, there is a different long-term trajectory of recovery. This is evident when subdividing athletes based on score, as those with CS2 > 0 had a mean *IE*_glob_ value significantly below that of controls at one year post-RTP, denoting increased inter-nodal path lengths and less efficient global information flow. The findings in the CS2 > 0 subgroup are also congruent with studies of more severe TBI, which reported lower global efficiency at the chronic phase of injury^32,34^, suggesting that the changes are a consequence of more severe pathophysiology, rather than imaging at a longer time interval post-injury. This is supported by a prior study in which mTBI patients with persistent symptoms had increased density and diversity of connections at 1–3 weeks post-injury, but were significantly decreased at 6 months, compared to controls and patients without persistent symptoms^33^. Nevertheless, we cannot yet definitively establish whether declines in *IE*_glob_ represent concussion pathophysiology itself or functional changes arising as an adaptive response to injury.

A more detailed investigation of nodal efficiency provides insights into the absence of concussion effects on *IE*_loc_. At acute injury, the mean *IE*_node_ value was unchanged between concussed and control groups, but the dispersion of nodal efficiency values was decreased, as areas of high *IE*_node_ were reduced and areas of low *IE*_node_ were increased. This further explains the observed *IE*_glob_ effects, indicating that the elevated acute values are due to greater clustering of isolated nodes, at the expense of connections between more densely connected nodes, resulting in a net increase in overall network efficiency that later dissipates at RTP. A reversed pattern of effect was also seen for the CS2 > 0 group at one year post-RTP, with increased dispersion of nodal efficiency values, driven by increases in areas of high *IE*_node_ and decreases in areas of low *IE*_node_. This mechanism of topological change may also generalize beyond concussion, as a study of normal aging in which reductions in *IE*_glob_ were larger than *IE*_loc_ also reported decreased nodal efficiency in frontal, temporal and subcortical areas^22^, i.e., within areas of low intrinsic nodal efficiency.

In terms of spatial localization, acute injury shows reliable *IE*_node_ decreases in regions of high intrinsic efficiency, including areas of the visual cortex, and increases in regions of lower intrinsic efficiency, including frontal and subcortical areas. One interpretation of this result is that the brain increases functional integration to support frontal/subcortical regions, which are vulnerable to compressive and shear forces after injury respectively^52,53^, at the expense of reduced efficiency in areas of visual processing. This reallocation of sensory resources may then contribute to the increased risk of re-injury shortly after concussion^54^. The present findings are also congruent with a recent study that reported suppression of BOLD scale-free dynamics in the visual cortex at acute injury^40^. Hence, the changes in BOLD dynamics may be an indicator of reduced nodal efficiency and reallocated functional resources within these regions. Interestingly, acute injury was associated with similar proportions of negative and positive effects on nodal efficiency, whereas CS2 was associated with predominantly positive effects. The long-term effects of concussion therefore appear to be more consistently driven by increased local efficiency in areas of visual processing, whereas long-term declines in nodal efficiency are not reliably observed.

Comparisons of network efficiency with other graph-theoretic measures yielded further information about the concussion-related changes in network topology. Among controls, both *IE*_loc_ and *IE*_glob_ had low-to-moderate correlations with other graph-theoretic measures, reinforcing that efficiency is a distinct aspect of network topology^18^. In terms of concussion effects, all graph-theoretic measures had comparable or lower effect sizes than *IE*_glob_, further confirming that concussion exerts relatively subtle effects on whole-brain network topology. Concussion effects were minimal for centrality measures of *ICB*_avg_ and *ICE*_avg_; these indices assess “hub-like” behaviour, in which highly-connected nodes facilitate the efficient flow of information through the network^55^. Although hubs are key to small-world network organization^56^, the present findings suggest that post-concussion changes in *IE*_glob_ are not driven by global changes in hub behaviour. The modularity index *IMOD* was congruent with *IE*_glob_, as both had elevated acute values with significant longitudinal declines in later sessions. The results suggest that changes in nodal efficiency tend to respect modular boundaries, with efficiency decreases (or increases) corresponding to reduced between-module (or increased within-module) connections. Degree assortativity showed the most long-lasting changes after concussion, with significant mean declines in *IDAS* from RTP to one year post-RTP. This indicates that, over the long-term, network nodes show increased tendency to connect with nodes of similar degree. An important feature of complex hierarchical networks, assortativity changes likely involve tradeoffs in network properties. For example, the lower assortativity values seen at acute injury and RTP are associated with global synchronization of neural networks^57^, whereas higher assortativity values seen at 1 year post-RTP are associated with greater network resilience to disruption^50^. The analysis of clinical covariates also showed significant associations between CS1 and measures of *ICB*_avg_ and *IMOD* at one year post-RTP, further suggesting divergent patterns of long-term brain recovery among athletes with differing clinical profiles.

Although this study provided new information about post-concussion changes in brain function, there are limitations which should be addressed in future work. The present study focused on network changes relative to RTP, but given the variability in days to RTP, this limits our ability to detect longitudinal changes that are unrelated to clinical recovery. A related issue is the large time interval between RTP and one year post-RTP. While none of the athletes had a second concussion in this interval, brain function in the latter session may influenced by other factors post-RTP, including exertional load^58,59^ and exposure to subconcussive impacts^12,60^. Given these limitations, further longitudinal studies may be informative, particularly if conducted at fixed time intervals post-injury and spanning multiple months post-injury. The long-term functional consequences of disrupted network efficiency are also unclear. Further research is needed to test for subtle neurocognitive deficits beyond the standard, largely symptom-based assessments of concussion. It must also be emphasized that changes in network efficiency reflect altered *statistical* relationships between brain regions, and do not necessarily correspond to changes in neuroanatomy or signalling pathways. It is an ongoing challenge to map functional connectivity to underlying brain physiology, although multi-modal MRI studies may provide further insights^28^. Lastly, this study was based on a fixed parcellation scheme. While it used adaptive spatial weights to mitigate the issues caused by using a pre-determined parcellation scheme^61^, future work should examine whether study findings generalize to other methods of brain parcellation, both predefined and data-driven.

This study provides new insights into the complex brain changes associated with long-term recovery after a concussion. Significant longitudinal changes in topological brain organization are seen from early injury to RTP, with a subset of individuals also showing significantly altered functional organization up to 1 year post-RTP. These findings provide evidence that concussion does not simply modulate the amplitude of functional connectivity, but also alters the organization of functional networks. Although the observed changes are modest, they provide evidence that indices of small-world behaviour may be an important aspect of concussion pathophysiology, particularly in understanding the heterogeneous clinical profiles and recovery times associated with concussion.

## Supplementary Information

Supplementary Information 1.

Supplementary Information 2.

Supplementary Information 3.

Supplementary Information 4.

## Data Availability

The authors have documented all data, methods, and materials used to conduct this research study, and anonymized data will be shared by request from any qualified investigator.
