# Supplementary material for: Long-term changes in the small-world organization of brain networks after concussion
Source: Sci Rep. 2021 Mar 25;11:6862. doi: 10.1038/s41598-021-85811-4 (PMC7994718; doi:10.1038/s41598-021-85811-4)
Supplement: Supplementary file 1 — Supplementary Information 1. [file 41598_2021_85811_MOESM1_ESM.docx]

**Supplemental File 1**: Athlete numbers by sport

**Table S1:** athlete numbers by sport, for both male (M) and female (F) groups, for N=167 controls and N=61 concussed athletes.

| **CONTROL** | **CONCUSSION** |
| --- | --- |
| Squash (1M)  Water polo (1M)  Lacrosse (10M* / 5F)  Basketball (3M / 10F)  Rugby (5M* / 11F*)  Football (11M*)  Soccer (15M / 10F)  Hockey (21M* / 30F)  Volleyball (13M / 21F) | Water polo (1F)  Basketball (1M / 2F)  Rugby (3M* / 6F*)  Football (4M*)  Hockey (3M* / 3F)  Volleyball (1M / 2F) |

* collision sports, defined as involving routine, purposeful body-to-body contact^47^
