# Supplementary material for: Long-term changes in the small-world organization of brain networks after concussion
Source: Sci Rep. 2021 Mar 25;11:6862. doi: 10.1038/s41598-021-85811-4 (PMC7994718; doi:10.1038/s41598-021-85811-4)
Supplement: Supplementary file 2 — Supplementary Information 2. [file 41598_2021_85811_MOESM2_ESM.docx]

**Supplemental File 2**: Estimation of community structure

For this paper, individual subject modularity (*MOD*) values were calculated on a fixed community partition, estimated from group data. This was achieved using the same subsample of 34/167 controls that were originally used to calculate parcel weights in *Methods: Graph theoretic analysis*. For this group, we have a set of $246\times246$ connectivity matrices $\boldsymbol{C}_{s}$ (*s*=1…34). These maps had the Fisher z-transform applied to ensure connectivity values were normally distributed, and were then averaged, producing $\boldsymbol{C}_{z,avg}$. We then applied the Infomap algorithm to obtain the optimal community partitioning as a function of percentile threshold *P*. We then examined community partitioning over the small-world regime of thresholds ranging from $P_{min}=3\%$ to $P_{max}$=26%, as identified in Figure 1.

We further applied two restrictions on the admissible range of thresholds. (1) Smaller *P* values create excessively sparse graphs, which tend to be erroneously fragmented into isolated single-node communities, therefore we identified smallest threshold $P_{min}'$ where on average, fewer than 5% of nodes isolated; this was assessed by running 100 bootstrap samples per threshold *P*, and calculating the average percentage of nodes with zeros edges. (2) larger *P* values create more globally connected networks, which cannot be partitioned into communities, therefore we identified largest threshold $P_{max}'$ where at least 2 communities are reliably identified; this was assessed by running 100 bootstrap samples per threshold *P*, and calculating the minimum number of partitions from the bootstrapped set of partitions.


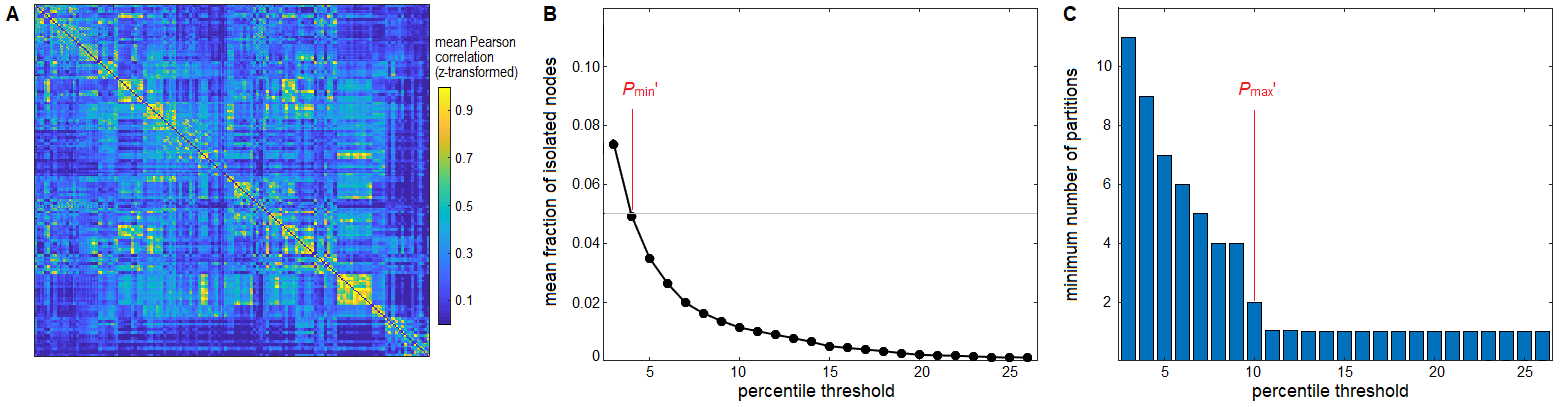


**Figure S1**: illustration of community partitioning approach. (A) group average of the Fisher z-transformed connectivity matrices. (B) mean fraction of the graph nodes that are isolated (i.e., zero edges) as a function of threshold, averaged over bootstrap resamples. (C) minimum number of graph partitions as a function of threshold, calculated as the minimum value over all bootstrap resamples.

From this set, we obtained new range $P_{min}'=4\%$ to $P_{max}$’=10%, from which we collected the set of 7 different community partitions. The consensus of these partitions was then obtained by using the consensus algorithm, producing the new robust average partition shown in Figure S2, consisting of 5 modules. They broadly correspond to mainly basal ganglia regions (module 1), occipital and medial parietal regions (module 2), medial temporal regions (module 3), prefrontal, cingulate and lateral parietal regions (module 4), along with insular, lateral temporal and sensorimotor regions (module 4). This partition was used to perform modularity analyses in the manuscript.


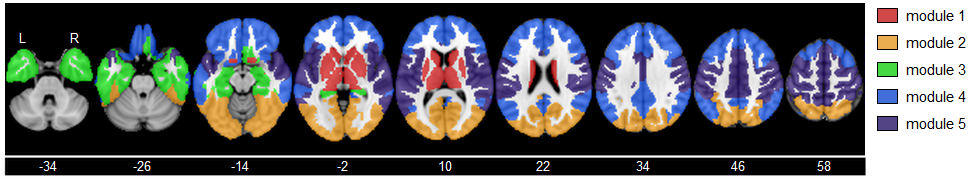


**Figure S2**: consensus community partition, consisting of 5 disjoint modules.
