# Supplementary material for: Long-term changes in the small-world organization of brain networks after concussion
Source: Sci Rep. 2021 Mar 25;11:6862. doi: 10.1038/s41598-021-85811-4 (PMC7994718; doi:10.1038/s41598-021-85811-4)
Supplement: Supplementary file 3 — Supplementary Information 3. [file 41598_2021_85811_MOESM3_ESM.docx]

**Supplemental File 3:** Nodal effect sizes

**Table S2**: summary of top 20 ROIs showing greatest absolute standardized effect sizes for ACU – CTL contrast, measured via bootstrap ratio (BSR). Brain regions are defined based on the Brainnettome Atlas (BNA).

|  | brain region | center of mass | | | bootstrap |
| --- | --- | --- | --- | --- | --- |
|  |  | (MNI coordinates) | | | ratio (BSR) |
| 1 | Superior frontal gyrus R (A6m) | 8 | -4 | 60 | -2.32 |
| 2 | Middle temporal gyrus R (A37dl) | 60 | -54 | 2 | -2.37 |
| 3 | Inferior parietal lobule L (A39c) | -34 | -80 | 30 | -2.02 |
| 4 | Medioventral occipital cortex L (rCunG) | -4 | -80 | 10 | -2.56 |
| 5 | Medioventral occipital cortex R (rCunG) | 8 | -76 | 10 | -2.21 |
| 6 | Medioventral occipital cortex R (vmPOS) | 14 | -64 | 12 | -2.76 |
| 7 | Lateral occipital cortex R (OPC) | 22 | -98 | 4 | -2.50 |
| 8 | Lateral occipital cortex L (lsOccG) | -22 | -78 | 36 | -2.13 |
| 9 | Thalamus R (rTtha) | 2 | -12 | 6 | -2.93 |
| 10 | Middle frontal gyrus L (A10l) | -26 | 60 | -6 | 2.80 |
| 11 | Middle frontal gyrus R (A10l) | 26 | 62 | -4 | 2.82 |
| 12 | Inferior frontal gyrus L (A44op) | -40 | 22 | 4 | 2.14 |
| 13 | Inferior frontal gyrus L (A44v) | -52 | 14 | 6 | 3.04 |
| 14 | Inferior temporal gyrus L (A20r) | -44 | -2 | -42 | 2.93 |
| 15 | Fusiform gyrus L (A20rv) | -32 | -16 | -32 | 2.50 |
| 16 | Parahippocampal gyrus L (A35/36r) | -26 | -6 | -34 | 2.21 |
| 17 | Parahippocampal gyrus L (TL) | -28 | -32 | -18 | 2.29 |
| 18 | Parahippocampal gyrus R (TL) | 30 | -30 | -18 | 2.55 |
| 19 | Hippocampus L (cHipp) | -28 | -30 | -10 | 2.37 |
| 20 | Hippocampus R (cHipp) | 30 | -28 | -10 | 3.08 |

**Key**: A6m= medial area 6, A37dl= dorsolateral area 37, A39c= caudal area 39 (PGp), rCunG= caudal cuneus gyrus, vmPOS= ventromedial parietooccipital sulcus, OPC= occipital polar cortex, lsOccG= lateral superior occipital gyrus, rTtha= rostral temporal thalamus, A10l= lateral area 10, A44op= opercular area 44, A44v= ventral area 44, A20r= rostral area 20, A20rv= rostroventral area 20, A35/36r= rostral area 35/36, TL= lateral posterior parahippocampal gyrus, cHipp= caudal hippocampus.

**Table S3**: summary of top 20 ROIs showing greatest absolute standardized effect sizes for 1YR(CS2>0) – CTL contrast, measured via boostrap ratio (BSR). Brain regions are defined based on the Brainnettome Atlas (BNA).

|  | brain region | center of mass | | | bootstrap |
| --- | --- | --- | --- | --- | --- |
|  |  | (MNI coordinates) | | | ratio (BSR) |
| 1 | Middle temporal gyrus L (A21c) | -66 | -30 | -12 | -2.72 |
| 2 | Precuneus L (A31) | -6 | -54 | 34 | -2.36 |
| 3 | Precuneus R (A31) | 6 | -54 | 34 | -2.54 |
| 4 | Middle frontal gyrus R (A9/46v) | 42 | 44 | 14 | 2.67 |
| 5 | Orbital gyrus R (A14m) | 6 | 48 | -6 | 2.52 |
| 6 | Inferior temporal gyrus R (A37elv) | 54 | -52 | -18 | 3.81 |
| 7 | Superior parietal lobule L (A7r) | -16 | -60 | 62 | 2.38 |
| 8 | Superior parietal lobule R (A7r) | 20 | -56 | 64 | 2.63 |
| 9 | Superior parietal lobule L (A7c) | -16 | -70 | 52 | 3.50 |
| 10 | Precuneus L (A7m) | -4 | -64 | 50 | 3.45 |
| 11 | Postcentral gyrus L (A1/2/3tonIa) | -56 | -14 | 16 | 2.48 |
| 12 | Postcentral gyrus R (A1/2/3tonIa) | 56 | -10 | 16 | 2.76 |
| 13 | Insular gyrus R (G) | 38 | -18 | 8 | 3.08 |
| 14 | Insular gyrus R (dIg) | 38 | -6 | 8 | 2.28 |
| 15 | Cingulate gyrus L (A24rv) | -4 | 8 | 26 | 2.28 |
| 16 | Cingulate gyrus L (A23c) | -8 | -22 | 40 | 3.57 |
| 17 | Medioventral occipital cortex R (rCunG) | 8 | -76 | 10 | 2.40 |
| 18 | Medioventral occipital cortex R (cCunG) | 8 | -90 | 12 | 3.26 |
| 19 | Lateral occipital cortex R (OPC) | 22 | -98 | 4 | 3.01 |
| 20 | Lateral occipital cortex L (msOccG) | -10 | -88 | 30 | 3.03 |

**Key**: A21c= caudal area 21, A31= area 31 (Lcl), A9/46v= ventral rea 9/46, A14m= medial area 14, A37elv= extreme lateroventral area 37, A7r= rostral area 7, A7c= caudal area 7, A7m= medial area 7 (Pep), A1/2/3tonIa= area 1/2/3(tongue and larynx), G= hypergranular insula, dIg= dorsal granular insula, A24rv= rostroventral area 24, A23c= caudal area 23, rCunG= rostral cuneus gyrus, cCunG= caudal cuneus gyrus, OPC= occipital polar cortex, msOccG= medial superior occipital gyrus.
