# Supplementary material for: Long-term changes in the small-world organization of brain networks after concussion
Source: Sci Rep. 2021 Mar 25;11:6862. doi: 10.1038/s41598-021-85811-4 (PMC7994718; doi:10.1038/s41598-021-85811-4)
Supplement: Supplementary file 4 — Supplementary Information 4. [file 41598_2021_85811_MOESM4_ESM.docx]

**Supplemental File 4**: Relationships with other nodal graph theoretic measures

In this file, comprehensive assessment of cross-sectional and longitudinal changes in integrated nodal efficiency (*IE*_node_) is provided, along with similar analyses for integrated nodal degree (*IDEG*_node_), betweenness centrality (*ICB*_node_) and eigenvector centrality (*ICE*_node_). Comparing the regional values of the mean control maps using Spearman correlations, with bootstrapped 95%CIs, nodal *IE*_node_ values show moderately strong associations with *IDEG*_node_ (ρ=0.591, [0.549, 0.631]) and with *ICE*_node_ (ρ=0.668, [0.637, 0.698]), but have minimal associations with *ICB*_node_ (ρ=-0.087, [-0.123, -0.062]). This relationship is also somewhat mirrored in the concussion effects analysis. For cross-sectional analyses, *IE*_node_, *IDEG*_node_ and *ICE*_node_ all show reduced occipital-parietal values and increased fronto-temporal values for ACU – CTL analysis, along with reduced occipital and cingulate values and increased lateral frontal values in subsequent RTP – CTL and 1YR – CTL analyses. For longitudinal analyses, some regions show similar effects between metrics, including reduced medial temporal and increased precuneal values at multiple time points, whereas others differ substantially. For example, anterior cingulate and inferior parietal regions show longitudinal increase for *IE*_node_ but decrease for *IDEG*_node_ and *ICE*_node_.


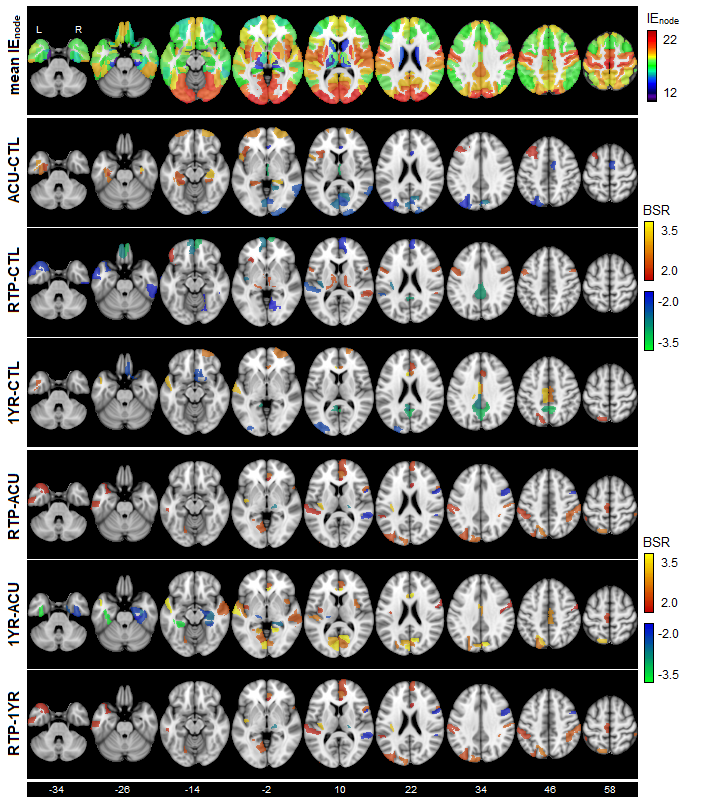


**Figure S3**: Effects of concussion on integrated nodal efficiency (*IE*_node_). Plots include (top panel) map of mean *IE*_node_ values, averaged over all controls; (middle panels) cross-sectional differences between concussed athlete values at acute injury (ACU), return to play (RTP) and one year post-RTP (1YR) relative to control values (CTL); longitudinal changes in concussed athlete values between imaging time points. Effect sizes are presented as bootstrap ratios (BSRs), with thresholding at |BSR|>2, equivalent to approximately *p*<0.05 uncorrected, and axial slices are labeled in MNI space coordinates.


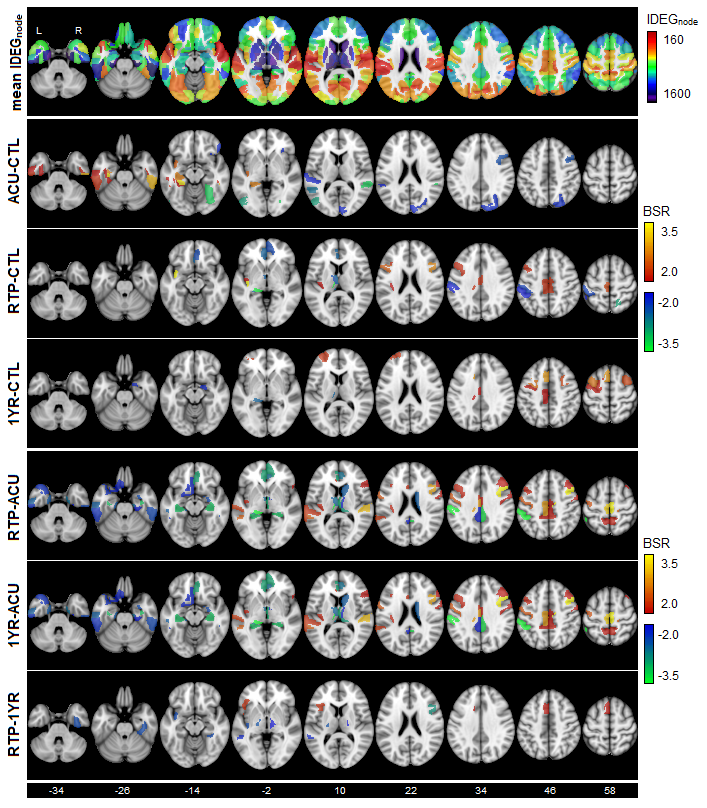


**Figure S4**: Effects of concussion on integrated nodal degree (*IDEG*_node_). Plots include (top panel) map of mean *IDEG*_node_ values, averaged over all controls; (middle panels) cross-sectional differences between concussed athlete values at acute injury (ACU), return to play (RTP) and one year post-RTP (1YR) relative to control values (CTL); longitudinal changes in concussed athlete values between imaging time points. Effect sizes are presented as bootstrap ratios (BSRs), with thresholding at |BSR|>2, equivalent to approximately *p*<0.05 uncorrected, and axial slices are labeled in MNI space coordinates.


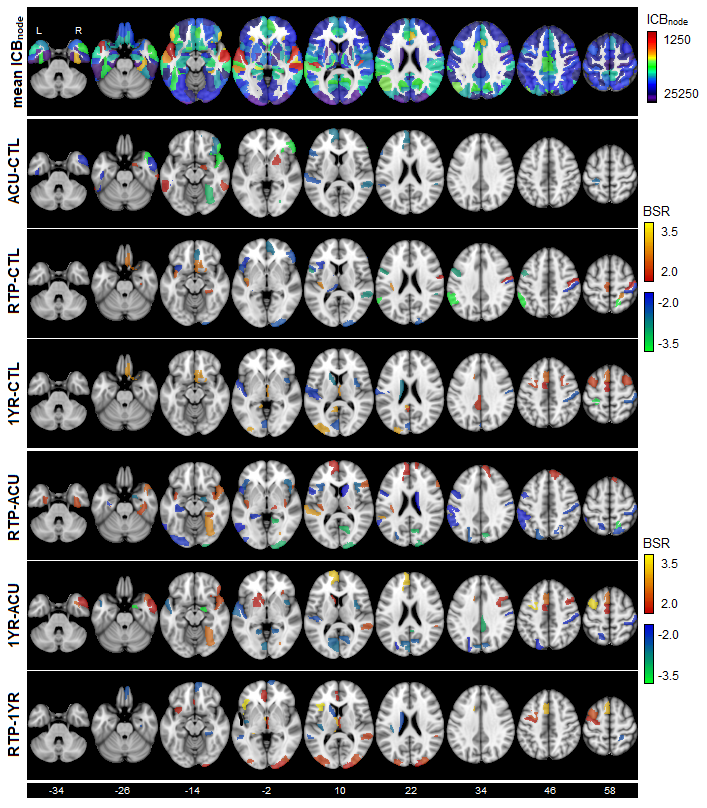


**Figure S5**: Effects of concussion on integrated nodal betweenness centrality (*ICB*_node_). Plots include (top panel) map of mean *ICB*_node_ values, averaged over all controls; (middle panels) cross-sectional differences between concussed athlete values at acute injury (ACU), return to play (RTP) and one year post-RTP (1YR) relative to control values (CTL); longitudinal changes in concussed athlete values between imaging time points. Effect sizes are presented as bootstrap ratios (BSRs), with thresholding at |BSR|>2, equivalent to approximately *p*<0.05 uncorrected, and axial slices are labeled in MNI space coordinates.


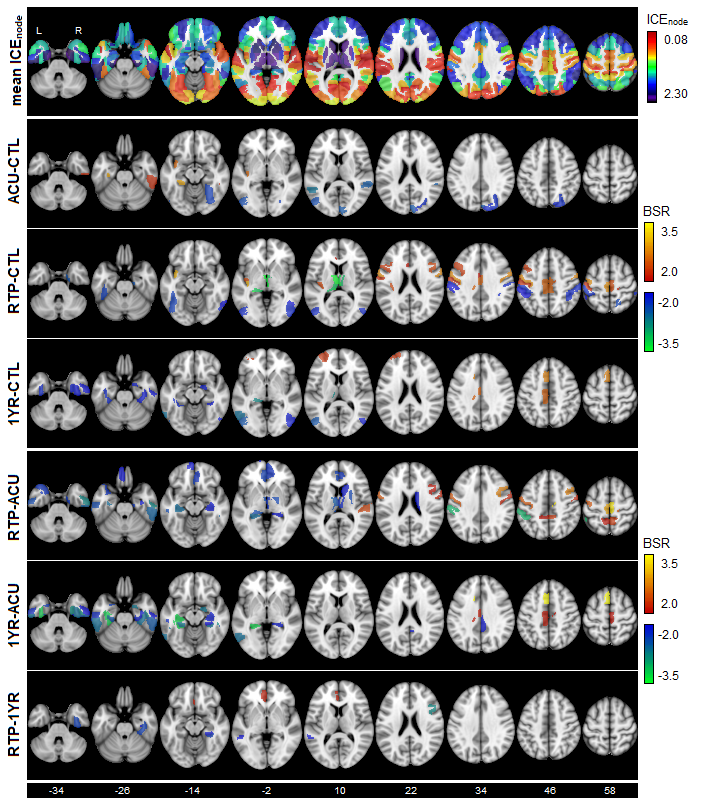


**Figure S6**: Effects of concussion on integrated nodal eigenvector centrality (*ICE*_node_). Plots include (top panel) map of mean *ICE*_node_ values, averaged over all controls; (middle panels) cross-sectional differences between concussed athlete values at acute injury (ACU), return to play (RTP) and one year post-RTP (1YR) relative to control values (CTL); longitudinal changes in concussed athlete values between imaging time points. Effect sizes are presented as bootstrap ratios (BSRs), with thresholding at |BSR|>2, equivalent to approximately *p*<0.05 uncorrected, and axial slices are labeled in MNI space coordinates.
